# Application of ultrasound in periodontics: Part I

**DOI:** 10.4103/0972-124X.44087

**Published:** 2008

**Authors:** Vive K. Bains, Ranjana Mohan, Rhythm Bains

**Affiliations:** 1 *Senior Lecturer, Department of Periodontics, Saraswati Dental College and Hospital, Lucknow (UP), India*; 2 *Professor and Head, Department of Periodontics, Saraswati Dental College and Hospital, Lucknow (UP), India*; 3 *Senior Lecturer, Department of Conservative Dentistry, Career PG Institute of Dental Sciences and Hospital, Lucknow (UP), India*

**Keywords:** Cavitation, microstreaming, real time images, sonochemicals, transducer, ultrasound

## Abstract

Ultrasonic is a branch of acoustics concerned with sound vibrations in frequency ranges above audible level. Ultrasound uses the transmission and reflection of acoustic energy. A pulse is propagated and its reflection is received, both by the transducer. For clinical purposes ultrasound is generated by transducers, which converts electrical energy into ultrasonic waves. This is usually achieved by magnetostriction or piezoelectricity. Primary effects of ultrasound are thermal, mechanical (cavitation and microstreaming), and chemical (sonochemicals). Knowledge of the basic and other secondary effects of ultrasound is essential for the development of techniques of application.

## INTRODUCTION

Ultrasound means sound that is not audible because it has frequencies above those of audible sound (30–20 KHz).[1] In 1880, Pierre and Jacques Curie discovered that crystals of many substances subjected to mechanical strains, develop electrical charges on their surface.[2] Soon as a natural corollary it was observed that when crystals of proper size or metals of proper configuration and content were subjected to an alternating electrical field, crystals or metals so treated, vibrated with oscillation of specific frequency and amplitude. In 1927, Wood and Loomis[3] published their work “*Physical and Biologic Effects of High Frequency Sound Waves of Great Intensity*”. Since that time, in medicine, ultrasound has been used mostly for treatment of neuromuscular and musculoskeletal ailments. First industrial use of magnetostrictive cutting device was to prepare cavities in synthetic sapphires for reception of gold inserts.[4] First use of this cutting method in dentistry was reported in Annals of Dentistry by Matthew C. Catuna in 1953.[5] Such instruments were used with abrasive slurry for preparation of tooth cavities prior to restoration.[6] With advent of high-speed drills, technology was repositioned for ultrasonics and power scaling in early 1960s, which revolutionized mechanical debridment.[7] Use of ultrasonic was first introduced in periodontal procedure in 1955 by Zinner[8] and have undergone many changes, and since then, simple compact devices have replaced large, heavy units. The single, bulky universal tip has been replaced by a variety of site specific, slimmer tips (some of which have been coined as microultrasonic).[9,10] Johnson and Wilson[11] reported adequate removal of calculus more rapidly with ultrasonic tips than with conventional scaling method. They also concluded that only light pressure is necessary to scale with ultrasonic.[11] Cementum under calculus is virtually unharmed,[12] comparatively little hemorrhage is associated with ultrasonic,[11] and patient reaction to the instrument especially in ANUG patients has been favorable.[12] In late 1980s and 1990s there was interest in nature of cleaning process where role of cavitation and acoustic microstreaming were shown to play role apart.[13,14] In addition to mechanical cavitational effects, ultrasonic treatment for tooth descaling also resulted in formation of sonochemical products.[15] This article is intended to review the basic principle and the effects of ultrasound.

## BASIC PRINCIPLE

**Figure 1:**
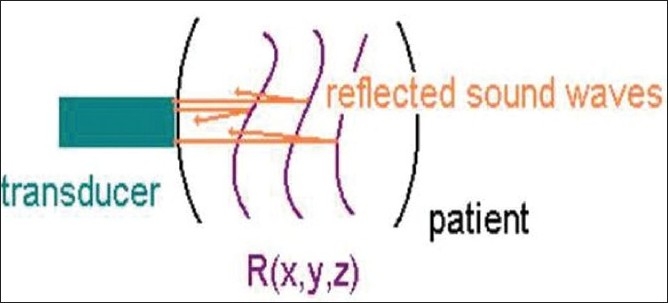
Diagram showing transmission and reflection of acoustic energy; A pulse is propagated and its reflection is received, both by the transducer

Ultrasonics is branch of acoustics concerned with sound vibrations in frequency ranges above audible level.[16–18] Ultrasound imaging, or ultrasound scanning or sonography, is a method of obtaining images from inside the human body through the use of high frequency sound waves.[16,18] As ultrasonic beam passes through or interacts with tissues of different acoustic impedence, it is attenuated by a combination of absorption, reflection, refraction, and diffusion.[18] The sound waves echoes are recorded and displayed as a real-time, visual image.[16,18] Ultrasound uses the transmission and reflection of acoustic energy.[16,17] A pulse is propagated and its reflection is received, both by the transducer,[16] a device which can convert electrical energy into sonic energy[18][Figure 1]. For clinical purposes, ultrasound is generated by transducers, which convert electrical energy into ultrasonic waves. This is usually achieved by magnetostriction or piezoelectricity.

Magnetostrictive devices undergo changes in their physical dimension when a magnetic field is applied to them. This is usually achieved by placing a ferromagnetic stack within a solenoid through which is passed a direct current. This produces stresses leading to a change in shape of the material. When an alternating current is passed through the solenoid the stack will then change its shape at twice the frequency of the applied magnetic field. Magnetostriction with a laminated ferromagnetic stack is used commonly in the design of ultrasonic scaling instruments, as it is a robust and easily manufactured system.[1] Magnetostrictive instruments operate between 18,000 and 45,000 cps, cps also known as Hertz, using flat metal strips in a stack or a metal rod attached to a scaling tip.[19] When an electrical current is supplied to a wire coil in the handpiece, a magnetic field is created around the stack or rod transducer causing it to constrict. An alternating current then produces an alternating magnetic field that causes the tip to vibrate. The tip movement of magnetostrictive units ranges from nearly linear, to elliptical or circular, depending on the type of unit, and shape and length of the tip.[19,20] Magnetostrictive tip movement allows for activation of all surfaces of the tip simultaneously, providing the option to use the side, back, or front of the tip for adaptation to the tooth surface.[19,20]

Piezoelectric system is based on the fact that certain crystalline structures such as quartz will be subject to a shape change when placed within an electrical field.[20] If an alternating voltage at an ultrasonic frequency is applied across a piezoelectric crystal, it will result in an oscillating shape change of the crystal at the frequency applied. This is then passed onto the working tip. Currently, the most widely used piezoelectric material is lead zirconate titanate (PZT).[18] Piezoelectric generators are more efficient at frequencies in the MHz rather than the KHz range, although some have been developed for use in dentistry. However, the crystalline structure has poor shock resistance and such instruments are more fragile than their magnetostrictive counterparts.[20] Piezoelectric unit operates in the 25,000–50,000 cps range and is activated by dimensional changes in crystals housed within the handpiece as electricity is passed over the surface of the crystals.[20] The resultant vibration produces tip movement that is primarily linear in direction, and generally allows only two sides of the tip to be active at any time.[19,20] Most current ultrasonic technology has advanced to include computer chips for regulating sustained power to the tip.[21]

## PROPERTIES OF ULTRASOUND

1. Ultrasound waves do not pass through air.[22]
2. Ultrasound has difficulty in penetrating bone and therefore can only see the outer surface of bony structures and not what lies within.[16]
3. Unlike X-rays, in which image is produced by transmitted radiation, the reflected portion of beam produces the image in ultrasonography.[18]
4. Ultrasound imaging is based on the same principles involved in the sonar used by bats, ships at sea, and anglers with fish detectors.[20] As the sound passes through the body, echoes are produced that can be used to identify how far away an object is, how large it is, its shape, and its consistency (fluid, solid, or mixed).[16]
5. Ultrasonograpy is a noninvasive and relatively inexpensive technique for imaging superficial tissues in real time.[16,17,22]
6. No ionizing radiation is involved in ultrasound imaging.[16,22]
7. Ultrasound waves have a nearly constant velocity of ∼1500 m/s in water. Sound wave velocity in water is similar to that in soft tissue.[16]

## BASIC EFFECTS OF ULTRASOUND

### Thermal effects

As a wave of ultrasound passes through tissues its energy is reduced and is dissipated as heat, leading to an elevation of tissue temperature. The effects of this on the tissues are dependent upon the size of temperature rise, the time over which it is maintained, and the thermal sensitivity of the tissue. In most tissues, the normal physiological response will be an alternation in the blood flow in the region due to reflex relaxation of the arterioles. The resultant increase in blood flow through the area will tend to control heating effects within a limited increase in temperature, with a temperature rise of less than 1°C resulting only in a minor overall increase in local metabolic rate. However, an excessive high temperature inevitably leads to tissue damage.[1,2]

### Cavitation

**Figure 2:**
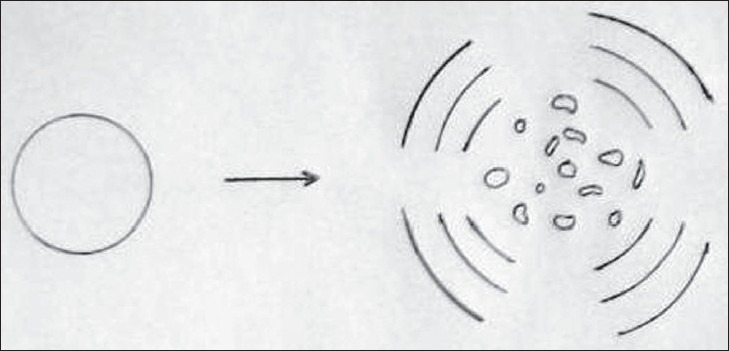
Diagrammatic representation of possible bubble collapse. A free buble collapsing to small fragments and radiateing shock waves. (Laird W.R.E. and Walmsley A.D, 1991)

**Figure 3:**
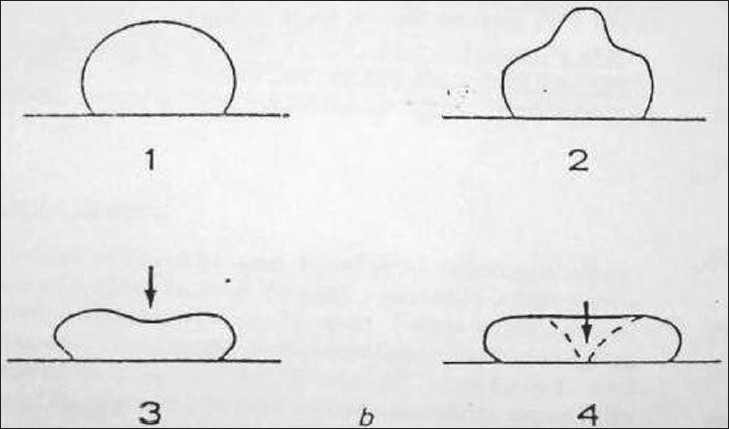
Diagrammatic representation of possible bubble collapse. (Laird W.R.E. and Walmsley A.D,1991). (1) Bubble on solid surface, (2) Undergoing deformation, (3) Producing a high velocity liquid jet, (4) Jet pierces bubble and damages solid surface

Cavitational activity in relation to ultrasound encompasses a continuous spectrum of bubble activity in a liquid medium. It ranges from gentle linear pulsation of gasfilled bodies in low amplitude sound fields (stable cavitation) to violent and destructive behavior of vapors-filled cavities (transient cavitation) in high amplitude sound fields.[1,23–25] The energy generated within these bubbles may result in shock waves or hydrodynamic shear fields which may disrupt biological tissues, and it is the production of these large disruptive forces which are of use in the removal of plaque and calculus during ultrasonic scaling.[1,26–28] The occurrence of cavitation requires the presence of gaseous bodies or bubbles in the medium which have been termed cavitation nuclei.[1,25] In the presence of an ultrasound field a bubble will grow and will undergo breathing pulsation in response to the applied pressure oscillations set up by the field.[1,24] As the bubble pulsates transverse waves are set up on its surface, which become distorted and unstable as the ultrasonic amplitude increases. Microbubbles will occur around the original bubble and will act as new sites for cavitational activity. Formation of microbubbles is associated with the onset of transient cavitation, where the bubbles show a ‘collapse’ phenomenon with the temperature of the gas in the bubble reaching thousands of degrees Celsius and several thousand atmospheres of pressure.[1,2,29] [Figures 2 and 3]

The demanding effects of transient cavitation are due to the shock waves radiated during the final stages of bubble collapse or high velocity liquid jets from nonlinear motions of the bubbles face. At low ultrasound frequencies in the order of 20–40 KHz growths of micronuclei and subsequent transient cavitation occur readily.[1]

Cavitation occurring in human blood can result in a thrombogenic effect and cause lysis of erythrocytes and platelets.[27] This may explain reduction in hemorrhage when using ultrasonic surgical instruments and dental scalers.[1,13]

### Acoustic microstreaming

**Figure 4:**
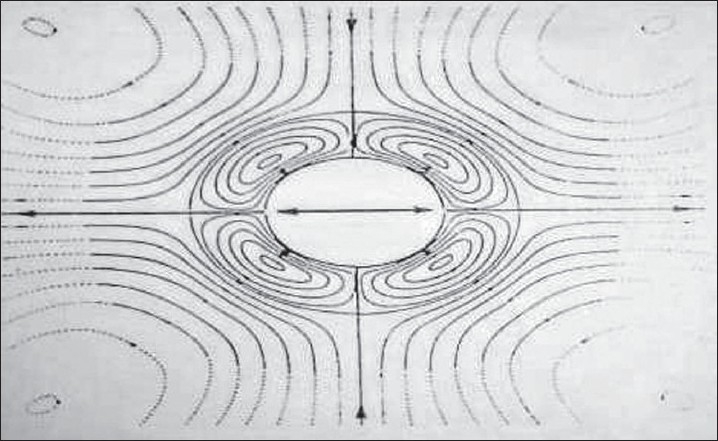
A theoretical prediction of acoustic microstreaming field generated around a solid cylinder oscillating within a stationary fluid (Laird W.R.E. and Walmsley A.D,1991)

**Figure 5:**
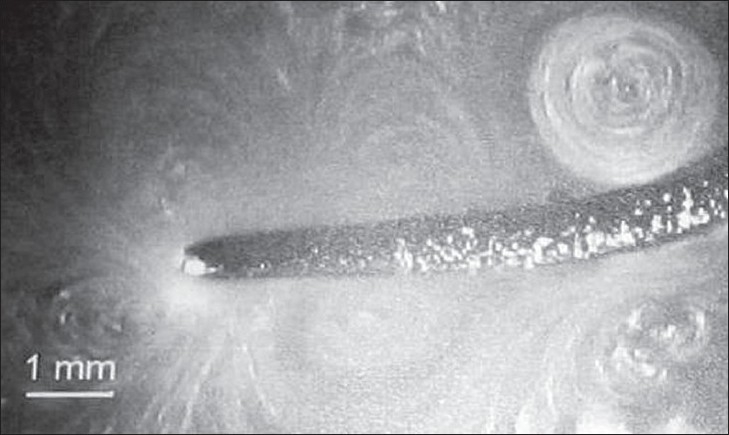
Video captured digitalized image showing acoustic microstreaming at 10.5μm displacement amplitude (Khambay B S, Walmsley A D; 1999)

**Figure 6:**
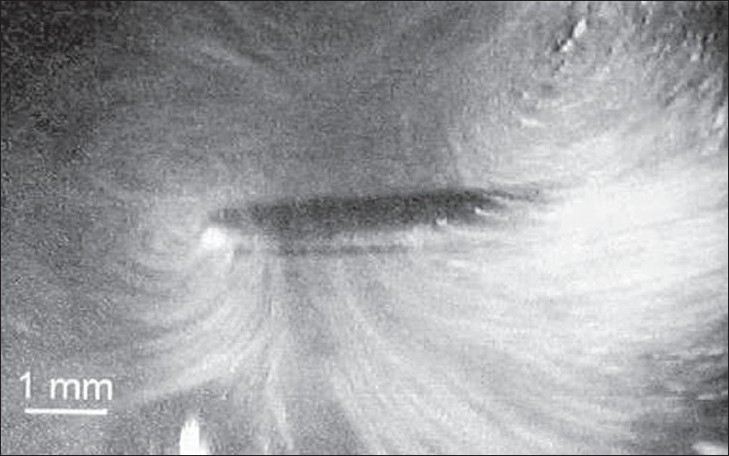
Video captured digitalized image showing acoustic microstreaming at 47.5μm different displacement amplitudes (Khambay B S, Walmsley A D; 1999)

The rapid cyclical volume pulsation of a gas bubble results in the formation of a complex steady state streaming pattern within the liquid close to the bubble surface.[1] Acoustic microstreaming is a phenomenon that exists in a fluid environment such as water and is characterized by the production of large shear forces.[30] It can be demonstrated around an oscillating solid cylinder within a fluid or a stationary cylinder within an oscillating fluid.[1] [Figure 4] Acoustic microstreaming occurring around ultrasonic scalers depends on displacement amplitude, tip orientation, and presence of water medium. It increases with increasing displacement amplitude, although it depends upon tip geometry, tip orientation, and distance from the oscillating tip.[30] [Figures 5 and 6]

The dimensions of the patterns demonstrate a rapid rate of change of streaming velocity with distance.[24] Therefore, although the velocities themselves are only of the order of a few centimeters per second,[25] the gradients due to the rate of change of velocity will produce large hydrodynamic shear stresses close to the oscillating object (i.e., probe or gas bubble) which may disrupt or damage biological cells or tissues.[1] Acoustic microstreaming may play a role in disruption of subgingival biofilms associated with periodontal diseases.[30] Acoustic microstreaming may also result in the disruption of blood flow and cells such as human platelets exposed to probes operating at 20 kHz (the level used in dentistry). At higher amplitudes, gelatinous aggregates of platelets can form an emboli resulting in possible blood vessel occlusion.[1,31]

### Chemical effects (sonochemicals)

In addition to mechanical cavitational effects, ultrasonic treatment for tooth descaling also resulted in formation of sonochemical products.[15] The agitation of ultrasonic vibrations releases ions contained in the propagating medium at great speed and intensity.[2] When ultrasonic cavitation (similar to ionizing radiation) acts on aqueous solutions of certain compounds, including dissolved air, oxygen, and nitrogen, free radicals produced due to water molecules decomposition reacts with these compounds or gases. Both free radicals and other compounds formed inside the solution (H_2_O_2_ or nitrous and nitric acids) are of particular biologic importance considering their chemical activities.[15] Free radicals produced are related to both displacement amplitude and the geometry of scaling tip.[32]

### Radiation forces

Any medium or object in the path of an ultrasonic beam is subjected to a radiation force, which tends to push the material in the direction of the propagating wave.[1,33] This force is small, but in a standing wave field may be enhanced and act over a short distance, so that dense particles in the medium are driven to regions of maximum acoustic pressure amplitude. In blood vessels, this may cause local aggregation of blood cells leading to stasis.[1,34] Radiation forces may also enhance cavitational activity within a standing wave field.[1,24]

## SECONDARY EFFECTS OF ULTRASOUND

The secondary effects of ultrasound are those responses, which may be elicited from or produced in a tissue during or following ultrasonic irradiation.

1. Vibrations of 25 KHz by frictional movement can be pressed directly against the tissue to produce coagulation.[2]
2. Gentle massage may produce a hyperemia with no tissue destruction, provided that the propagating medium is flowing continuously between the tool and the tissue.[2]
3. Ultrasound applied to tissues of high fluid content will evoke bubble formation or degassing within tissue (cavitation).[2]
4. Tissue turgid with fluid or frozen solid may be cut with facility with ultrasonic instruments of proper design and frequency (tissue surgery).[2,35]

### Other tissue effects

1. Research workers have demonstrated that the application of high frequency vibrations has helped improve myalgia and tendon extensibility.[2]
2. In medicine it has been shown that scar tissue, particularly that resulting from burns, may be softened following the use of ultrasound. The fibrotic gingiva of chronic gingivitis, being a type of scar tissue, was subjected to ultrasound shows similar results to those described.[2]
3. Rubbing or pressing a vibrating tool tip against soft tissue coagulates the surface and produces a form of soft tissue curettage. Such curettage may be performed within the crevice or on the buccal or labial aspects of the gingiva.[2]
4. When applied to gingiva in experimental animals, ultrasonic vibrations disrupt tissue continuity, lifting off epithelium, dismembering collagen bundles, and alter the morphology of fibroblast nuclei.[36] Ultrasonic vibrations directed at tissue interfaces, that is, the epithelium-connective tissue junction, spread laterally lifting off the epithelium. The connective tissue below is dehydrated and the collagen bundles are mechanically pushed apart. The defect thus created in the tissues is a form of coagulated wound.[2]
5. In addition to soft tissue curettage, ultrasound may be used for gingival surgery. Periodontal curettes sharpened to a razor edge and activated with ultrasonic vibrations are able to excise gingival tissue.[2]

## CONCLUSION

Ultrasonic is branch of acoustics concerned with sound vibrations in frequency ranges above audible level, has been used in dentistry since 1950s. The understanding of its basic principles and properties allow us to consider more fully, the effectiveness, safety, limitations, and rationale of ultrasound in dentistry.
